# Temperature effects on the tympanal membrane and auditory receptor neurons in the locust

**DOI:** 10.1007/s00359-014-0926-y

**Published:** 2014-07-22

**Authors:** Monika J. B. Eberhard, Shira D. Gordon, James F. C. Windmill, Bernhard Ronacher

**Affiliations:** 1 Department of Biology, Behavioural Physiology Group, Humboldt-Universität zu Berlin, Invalidenstrasse 43, 10115 Berlin, Germany; 2 Department of Electronic and Electrical Engineering, Centre for Ultrasonic Engineering, University of Strathclyde, Royal College Building, 204 George Street, Glasgow, G1 1XW UK; 3 Present Address: Department of Biological Sciences, Dartmouth College, 78 College Street, Hanover, NH 03755 USA

**Keywords:** Temperature, Tympanal membrane, Auditory receptor neurons, Hearing, Insect

## Abstract

Poikilothermic animals are affected by variations in environmental temperature, as the basic properties of nerve cells and muscles are altered. Nevertheless, insect sensory systems, such as the auditory system, need to function effectively over a wide range of temperatures, as sudden changes of up to 10 °C or more are common. We investigated the performance of auditory receptor neurons and properties of the tympanal membrane of *Locusta migratoria* in response to temperature changes. Intracellular recordings of receptors at two temperatures (21 and 28 °C) revealed a moderate increase in spike rate with a mean Q10 of 1.4. With rising temperature, the spike rate–intensity–functions exhibited small decreases in thresholds and expansions of the dynamic range, while spike durations decreased. Tympanal membrane displacement, investigated using microscanning laser vibrometry, exhibited a small temperature effect, with a Q10 of 1.2. These findings suggest that locusts are affected by shifts in temperature at the periphery of the auditory pathway, but the effects on spike rate, sensitivity, and tympanal membrane displacement are small. Robust encoding of acoustic signals by only slightly temperature-dependent receptor neurons and almost temperature-independent tympanal membrane properties might enable locusts and grasshoppers to reliably identify sounds in spite of changes of their body temperature.

**Electronic supplementary material:**

The online version of this article (doi:10.1007/s00359-014-0926-y) contains supplementary material, which is available to authorized users.

## Introduction

Temperature affects many functions in poikilothermic (ectothermic) animals, as their body temperature changes with ambient conditions. Chemical reactions necessary to maintain life-sustaining processes depend on temperature—for example, temperature shifts modulate basic properties of nerve cells such as spike rate, conduction velocity, and spike amplitude (Burrows 1989; Janssen 1992; Franz and Ronacher 2002; Robertson and Money 2012). Thus, ambient temperature affects poikilothermic animals’ behavioural performances including locomotion (Bennett 1990; Full and Tullis 1990; Charabidze et al. 2008), feeding (Hückesfeld et al. 2011) or communication (von Helversen 1972; Heller 1986; Bauer and von Helversen 1987; Hoy 1992). The sensory and motor systems of many poikilotherms often need to function effectively over a wide range of temperatures (Robertson 1993), as they occur in various habitats characterized by large temperature changes during the course of a day or the cycle of a year. Large temperature differences may also occur at a microclimatic level, e.g. in a meadow where they may total up to 10 °C between the ground and the top of vegetation (Römer 2001).

The ability to detect and process sounds is an important feature in many insects, enabling them to identify predators, prey, or mating partners. Both the production and perception of acoustic signals depend on body temperature, as the properties of muscles and neurons involved change with temperature shifts (Heller 1986; Bauer and von Helversen 1987). Calling songs of various insects such as cicadas, planthoppers, crickets, katydids, grasshoppers, and drosophila change with ambient temperature (for review see Sanborn 2006). Receivers of acoustic signals are also affected, since spike rates and sometimes sensitivity of auditory neurons increase, and temporal resolution is improved with rising temperature (Wolf 1986; Oldfield 1988; Coro et al. 1994; Franz and Ronacher 2002; Prinz and Ronacher 2002; Fonseca and Correia 2007; Korsunovskaya and Zhantiev 2007). Therefore, without adjustment to temperature changes, insects would only be able to reliably produce and perceive acoustic signals in a small temperature range, which would limit the usefulness of sounds as communication signals (Sanborn 2006).

Locusts are commonly used in studies including olfaction (e.g. Raman et al. 2010; Martin et al. 2011; Joseph et al. 2012), vision (e.g. Guest and Gray 2006; Simmons et al. 2010; McMillan and Gray 2012), and neuronal processing of auditory stimuli (e.g. Neuhofer et al. 2008; Clemens et al. 2011; Wohlgemuth et al. 2011; Neuhofer and Ronacher 2012). However, less is known regarding how their ears or tympanal membranes respond to sound under different temperature conditions.

**Fig. 1:**
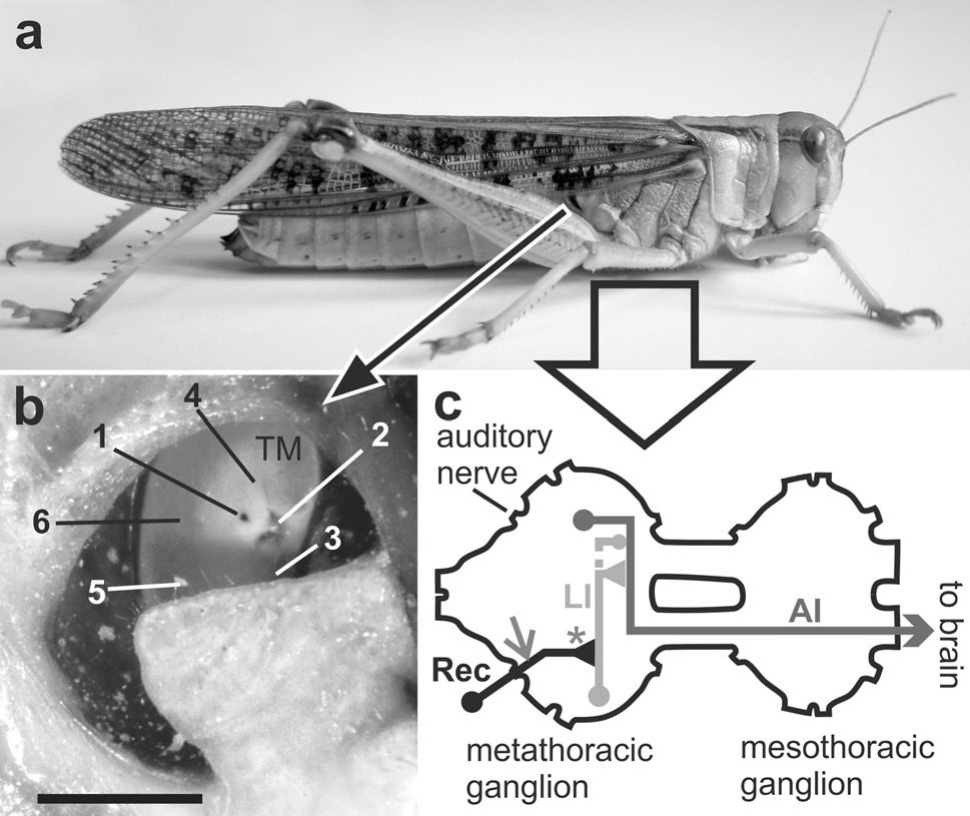
Scheme of the peripheral auditory pathway of *Locusta migratoria*. **a** The ears (tympana) are located laterally on the first abdominal segment. **b** Six representative locations on the tympanal membrane (TM) were chosen to measure movement of the membrane during acoustic stimulation. Points 1–4 represent attachment points of Müller’s organ, the auditory sensory organ. *Bar* 1 mm. **c** Auditory receptor neurons (Rec), attached to the tympanal membrane, deliver afferent signals via their axons to the metathoracic ganglion where they project onto local interneurons (LI), which connect to ascending interneurons (AI). Receptor neurons were recorded intracellularly within the metathoracic ganglion (*grey* *arrow* and *asterisk*)

In the present study we investigate the effects temperature exerts on the tympanal membrane properties and performance of auditory receptor neurons of *Locusta migratoria* L. The ears of grasshoppers and locusts are located laterally on the first abdominal segment (Fig. 1a). Sound induces a unidirectional travelling wave in the tympanal membrane, by which energy of different frequencies is channelled to distinct attachment sites of correspondingly tuned receptor neurons, peaking at point 1 (Fig. 1b, Online Resource 1). Thus, the tympanal membrane serves to both receive sound and differentiate its frequency content (Windmill et al. 2005, 2008), before receptor neurons deliver afferent signals to the metathoracic ganglion (Fig. 1c). Here, important first processing steps take place before further transmission to the brain (Stumpner et al. 1991; Clemens et al. 2011).

We recorded intracellularly from receptor neurons within the metathoracic ganglion while the temperature of the preparation was lowered from 28 °C to 21 or 22 °C. From the spike rate–intensity–functions, temperature coefficients (Q10) were calculated. Data on receptor neuron spike rate have also been used in another study focussing on theoretical considerations (Roemschied et al. 2014). To investigate whether temperature-dependent changes in the physical movement of the tympanal membrane contribute to the observed changes in receptor responses, the tympanal membrane was monitored at six representative locations, using a microscanning laser Doppler vibrometer.

## Materials and methods

### Animals

**Fig. 2:**
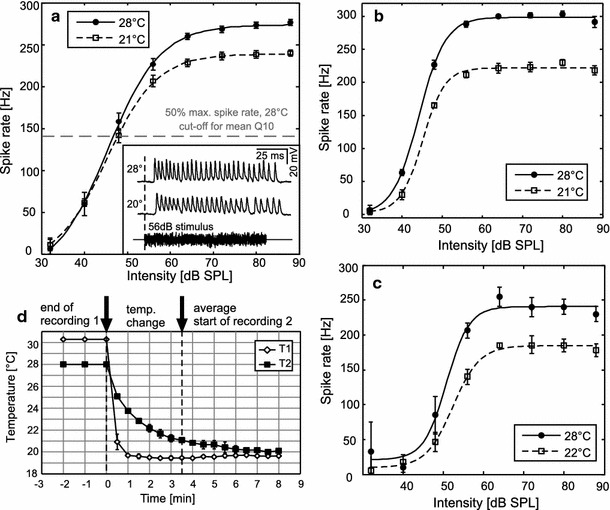
Spike rate–intensity–functions of three receptor neurons recorded intracellularly each at two different temperatures (only ipsilateral acoustic stimulation shown). **a** The receptor neuron shows a typical sigmoidal spike rate–intensity–function, with a dynamic range between ca. 35 and 60 dB SPL, saturating at 70 dB SPL. The cutoff for calculation of mean Q10 values was based on spike rates above 50 % maximum at the warmer temperature (*grey dashed line*, see “Materials and methods”). *Inset* acoustic stimulus (lower trace) and voltage traces of the recording, showing shorter action potential durations at the higher temperature. **b** Spike rate–intensity–functions for a high-frequency receptor and **c** a low-frequency receptor. A Boltzmann function was fitted to the data points (see “Materials and methods” and Fig. 3a for definitions). **d** Calibration curves of temperature measured directly at the Peltier element (T1) and at the inner side of the tympanal membrane, as close as possible to the attachment sites of the receptor neurons (T2). For all temperature measurements during intracellular recordings, the temperature was calibrated according to T2; thus, temperature ranges from 6 to 8 °C could be acquired. We adhered to a conservative approach: the temperature of the preparation was derived from the calibration curve at the onset of a recording (lasting 40 s). Although temperature may still have been subject to small changes during the recording, this procedure ensured that temperature changes were, if at all, slightly underestimated

Adult *L. migratoria* were obtained from commercial suppliers (Hellweg, Germany; Blades Biological Ltd., UK). Animals were housed gregariously at 22–25 °C with ad libitum food supply (organic lettuce, oats). Temperature regulation for the trials was obtained by placing the animals directly onto a Peltier element (3 cm × 1.5 cm) connected to a 2 V battery and a potentiometer. For the electrophysiology experiments, temperature was monitored and recorded with a digital thermometer (GMH 3210, Greisinger electronic GmbH, Regenstauf, Germany) connected to an NiCr–Ni-thermoelement (GTF 300, Type K, Greisinger electronic GmbH, Regenstauf, Germany). To prevent any disturbances by the thermoelement during recordings, it was fixed between the Peltier element and the preparation. In four control preparations, temperature was measured by two thermoelements, one fixed between the Peltier element and the preparation and one inside the preparation, placed as close as possible to the inner side of the tympanum, where the receptor neurons attach. From these temperature measurements, a calibration curve was established and subsequently used to correct the temperature measured during recordings. The temperature differences between high and low temperature treatment were on average 6.5 ± 0.8 °C (mean ± SD; Fig. 2d). In the laser vibrometry experiments we used an infrared thermometer (IR 650-12D, Voltcraft, UK) pointed directly at the tympanum.

### Sound stimulation

Broadband noise stimuli (100 ms duration, 1–40 kHz electrophysiology, and 1–30 kHz laser vibrometry) were created with Matlab (R2011a, The MathWorks Inc., USA) followed by a 100 kHz D/A conversion (BNC-2090A; National Instruments, Austin, TX, USA). An intensity range from 32 to 88 dB SPL was covered in 8-dB steps for electrophysiology; for laser vibrometry, two intensities were used (74 and 88 dB SPL). In the electrophysiological experiments, the stimulus sound was routed through a computer-controlled attenuator (ATN-01M; npi electronic GmbH, Tamm, Germany) and an audio amplifier (Pioneer stereo amplifier A-207R, Pioneer Electronics Inc., USA). Acoustic stimuli were broadcast unilaterally by speakers (D2905/970000; Scan-Speak, Videbæk, Denmark) located at ±90° (left and right side of the insect) and 30 cm from the preparation. In the electrophysiological recordings, sound was played first either from the right speaker and then from the left speaker or vice versa to obtain a directional characteristic for each receptor neuron. Sound intensity was calibrated with a half-inch microphone (type 4133; Brüel & Kjær, Nærum, Denmark) and a measuring amplifier (type 2209; Brüel & Kjær, Nærum, Denmark), positioned at the site of the preparation. For the laser vibrometry experiments, the stimulus sound was routed through the laser vibrometer computer, amplified (TA-FE370, Sony, Japan), and played with the speaker (Air-Motion Transformer, Heil, USA) positioned facing the tympanum. A microphone (type 4138, Brüel & Kjær, Denmark) connected to the laser software was placed at the location of the tympanum to calibrate the sound pressure.

### Electrophysiology

Legs, wings, head, and gut were removed from adult *L. migratoria* before the animals were fixed with a thin layer of wax, dorsal side up, onto the Peltier element attached to a holder. The thorax was dissected dorsally to expose the metathoracic ganglion, which was subsequently stabilized on a small NiCr platform. The thoracic cavity was filled with locust saline solution (Pearson and Robertson 1981). Auditory receptors were recorded intracellularly in the metathoracic ganglion, near the auditory nerve (Fig. 1c, arrow), or in the frontal auditory neuropil (Fig. 1c, asterisk), using glass microelectrodes (borosilicate, GC100F-10; Harvard Apparatus, Edenbridge, UK) filled with a 3–5 % solution of Lucifer yellow in 0.5 M LiCl. All electrophysiological experiments were carried out in a Faraday cage lined with foam prisms to minimize echoes. Neural responses were amplified (BRAMP-01; npi electronic GmbH, Tamm, Germany) and recorded by a data-acquisition board (BNC-2090A; National Instruments, Austin, TX, USA) with a sampling rate of 20 kHz. Acoustic stimuli as well as intracellular recordings were stored digitally by a custom-made programme (LabView 7 Express, National Instruments, Austin, TX, USA). Each measurement was repeated five times per intensity and stimulation side (ipsi- and contralateral). In most experiments, recordings were conducted first at the high temperature (28 °C), then the preparation was cooled down to 21 °C and the stimulation programme was repeated for the same neuron at the low temperature. The temperature change at the Peltier element was completed in approximately 1 min (see Fig. 2d). The stimulation programme started after another 1–2 min. After completion of the recordings, Lucifer Yellow was injected into the recorded cell by applying hyperpolarizing current. Subsequently, the thoracic ganglia were removed, fixed in 4 % paraformaldehyde, dehydrated, and cleared in methyl salicylate. The stained cells were identified under a fluorescent microscope according to their characteristic morphology (Römer and Marquart 1984; Halex et al. 1988; Stumpner and Ronacher 1991). Ipsi- and contralateral stimulation sides were defined relative to the axon of the receptor neuron.

A total of nine receptor neurons in eight preparations were recorded; in one preparation, two receptor neurons were recorded but treated as independent measurements, as there was at least 15 min between recordings, new electrodes had been used, and the site of recording had been changed from left to right. The recorded receptor was checked to determine if it was a high-frequency receptor or a low-frequency receptor by applying 5 and 15 kHz stimuli in four of the recordings. In our sample, no difference in reaction to temperature was found between the recordings of two high-frequency receptors (Fig. 2b) and two low-frequency receptors (Fig. 2c).

**Fig. 3:**
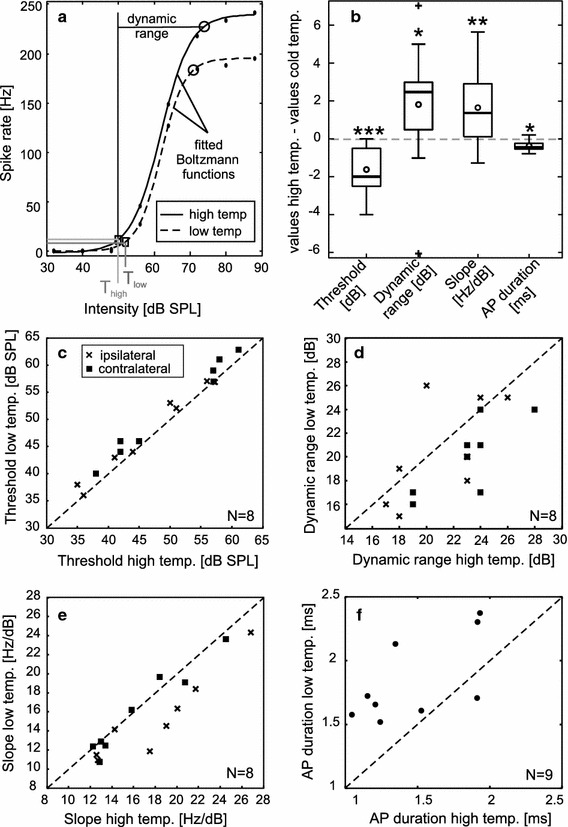
**a** A Boltzmann function was fitted to the spike rate–intensity–functions; thresholds at high (*T*_high_) and low temperature (*T*_low_) were defined as the intensities where spike rate rose above the mean spontaneous background spike rate plus one standard deviation. The dynamic range was measured from threshold to 95 % maximum spike rate. **b** Changes in firing threshold, dynamic range, and slope of the sigmoidal function (*N* = 8, ipsi- and contralateral stimulation), as well as action potential duration (*N* = 9) were determined by subtracting the values at the cold temperature (temp.) from the values at the warm temperature (temperature spans between 5 and 8 °C). *Box plots* show median (*thick horizontal lines*), mean (*circles*), quartiles (*box*), 1.5 IQR (*whiskers*), and outliers (+). Significances were determined using Wilcoxon matched pairs signed-rank tests. **c**–**f** Pairwise comparisons of firing threshold **c**, dynamic range **d**, slope **e**, and action potential (AP) duration **f** at the two temperatures (temp.)

Spike times were extracted from the digitized recordings by applying a voltage threshold. Mean spike rates were calculated for each intensity to obtain spike rate–intensity–functions per neuron, stimulation side, and temperature. From these curves, temperature coefficients (Q10 values) of the firing rate were determined using Eq. 1,

1 $${\text{Q}}10 = \left( \frac{X}{Y} \right)^{{\frac{10}{(Tx - Ty)}}} ,$$

where *X* is the spike rate at the higher temperature (*Tx*), and *Y* is the spike rate at the lower temperature (*Ty*). Thus, a Q10 value above 1 implies a greater spike rate at the higher temperature, while a Q10 = 1 indicates that the spike rates did not differ. For each recorded neuron, a mean Q10 value per stimulation side was calculated using only values with spike rates above 50 % maximum spike rate at the high temperature (see Fig. 2a). This was done to omit sub-threshold or near-threshold data, which would have resulted in unreliable Q10 values. To establish if the sensitivity of the receptors changes with temperature, we determined the firing threshold for each spike rate–intensity–function by fitting a sigmoidal Boltzmann function (Eq. 2) to each of the plotted graphs (Figs. 2, 3a),

2 $$y = \frac{p(1)}{{1 + {\text{e}}^{ - (x - p\left( 2 \right))/p\left( 3 \right)} }},$$

where *p*(1) is the maximal spike rate, *p*(2) is intensity in dB SPL at half-maximal spike rate, and *p*(3) is the slope of the function.

Threshold was defined as the intensity where the fitted function rose above the mean spontaneous background spike rate plus one standard deviation. The dynamic range of a receptor neuron between threshold and saturation of the sigmoidal function is important for the animal’s ability to discriminate between sound intensities. The steeper the slope of the function, the better is the intensity resolution of the receptor cell. To investigate if the dynamic range of receptor neurons depends on temperature, the slopes of the sigmoidal functions were also compared and the width of the dynamic range was determined from threshold to 95 % maximum spike rate (Fig. 3a). For one receptor, threshold, slope and dynamic range could not be reliably calculated as firing started in response to the lowest stimulus intensity (therefore: *N* = 16(8) instead of 18(9) for those values; two values per receptor neuron; ipsi- and contralateral stimulation). The shape of action potentials was investigated by superimposing all spontaneously emitted spikes (spikes during acoustic stimulation were not considered to avoid any changes in action potential shape due to high spike rates) and calculating a mean action potential shape per neuron and temperature (custom-made Matlab function by Frederic Roemschied). From the mean shape, action potential duration was measured at half-maximum amplitude. Significance of differences between high and low temperature in threshold, slope, width of dynamic range, and action potential duration was estimated using Wilcoxon matched pairs signed-rank tests. All analyses were done using Matlab (R2012a, The MathWorks, USA), and subsequent graphs were edited in CorelDraw (X6, Corel Corporation, Canada).

### Laser vibrometry

Wings were cut to allow direct access to the tympanal membrane for the laser measurements (Polytec PSV-300-F, Germany, with an OFV-056 scanning head fitted with a close-up unit attachment). Animals were firmly attached, ventral side down, to the Peltier element using BLU-TACK (Bostik-Findley, Stafford, UK) and were given 1–2 min to adjust to temperature (20 or 31 °C) before the sound was played. First, the entire membrane was scanned, and then six representative points (see Fig. 1b) were individually measured, to achieve the greatest accuracy. In some individuals, not all points could be measured, mainly because of the cuticular structure covering the tympanum (see Fig. 1b); this was especially true for point 3. Each laser measurement was averaged over 15 repeats. After completion of a recording at one temperature, the whole laboratory and Peltier element were heated up or cooled down and measurements were repeated at the warmer/cooler temperature. It took approximately 10 min to heat/cool the small laboratory. Gain data between the laser and the microphone were used to account for any sound volume variations, and data were considered of sufficient quality when coherence exceeded 85 % (see Windmill et al. 2005). Displacement gain values were obtained for every 12.5 Hz and pooled together in 500 Hz groupings.

Data were analysed in two ways: (1) identifying if membrane displacement was greater for either temperature and (2) obtaining the Q10 value. Membrane displacement differences were determined from the displacement values per frequency (58 frequency bins) per point (six points, see Fig 1b), per animal (*N* = 31). We calculated the percentage of animals whose membrane moved more in warm conditions (31 °C) by dividing the warm values with the cold values where a ratio of greater than one indicated more movement in the warm temperatures. We then analysed the percentage that moved more with a Chi-squared test for each of the six points and all points pooled together (one mean value per individual over all six points). Q10 values were calculated in a similar fashion as the electrophysiology (Eq. 1), where *X* was the amplitude gain at the higher temperature (*Tx*) and *Y* was the gain at the lower temperature (*Ty*), thereby the electrophysiology and laser physiology results were comparable.

## Results

### Receptor neurons

Intracellular recordings at 28 and 21 °C revealed that spike rate increased with increasing temperature (Fig. 2). The spike rate changes corresponded to an average Q10 of 1.40 ± 0.21 (mean ± SD; the range of observed Q10 values was 1.19–1.82). All receptor neurons exhibited a sigmoidal spike rate–intensity–function; the dynamic range of most receptors started at 35–40 dB SPL and saturated between 60 and 70 dB SPL (see three examples in Fig. 2a–c). With increasing temperature, thresholds decreased by 1.63 dB on average (Fig. 3b, c). This change was significant in a pairwise comparison (Wilcoxon matched pairs signed-rank test, *W* = 0, *N* = 16, *P* < 0.001). The dynamic range increased by ~1.8 dB (Wilcoxon matched pairs signed-rank test, *W* = 19, *N* = 16, *P* < 0.05; Fig. 3b, d), and the slopes of the sigmoid functions became marginally steeper with increasing temperatures (Wilcoxon matched pairs signed-rank test, *Z* = −2.74, *N* = 16, *P* < 0.01; Fig. 3b, e). With higher temperature, the duration of action potentials decreased by 0.06 ms per 1 °C on average (Wilcoxon matched pairs signed-rank test, *W* = 2, *N* = 9, *P* < 0.05; Fig. 3b, f).

### Tympanal membrane

**Fig. 4:**
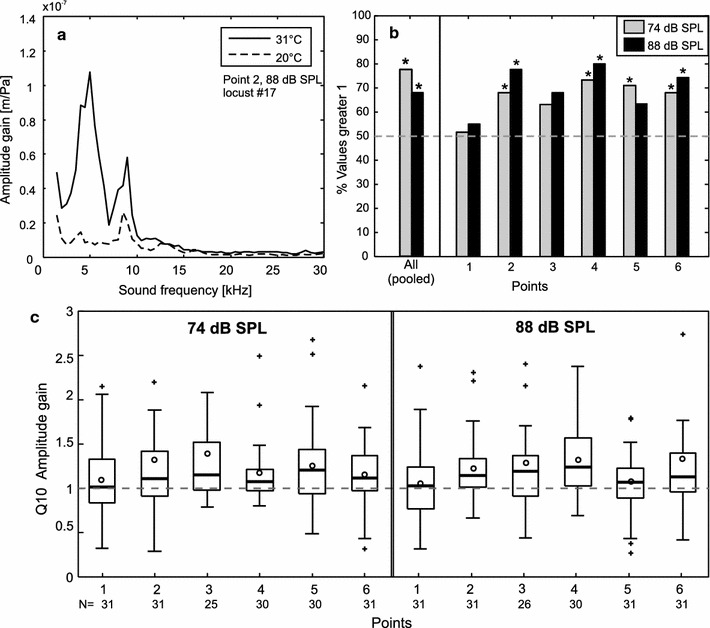
Displacement of the tympanal membrane at two different temperatures. **a** Example of recorded amplitude gain per sound frequency bin measured at point 2 of the tympanal membrane for one individual at 31 and 20 °C. **b** Percentage of animals with tympana that moved more with warmer temperatures for each measured point and combined points. *Asterisks* identify significance (Chi-squared tests; *P* < 0.05), *gray dashed line* indicates 50 % of individuals. **c** *Box plots* of Q10 values of tympanal membrane movement for all measured points and the two sound intensities used. Note that in all points, median (*line*) and mean (*circle*) Q10 values lie above 1

Tympanal displacement followed an expected frequency response and travelling wave pattern at both temperatures (20 and 31 °C) (Fig. 4a, Supplementary material 1a). Across frequencies, the tympanal membrane moved more with warmer temperatures, as indicated by the values remaining above 1 when hot data was divided by cold data (Fig. 4b). Pooling frequency data together for each measured point and individual, the amplitude gain increased significantly for all points together irrespective of stimulus intensity (all points combined: Chi-square test, 74 dB SPL: *χ* ^2^ = 9.32, *P* < 0.01; 88 dB SPL: *χ* ^2^ = 3.90, *P* < 0.05, *N* = 31), and for 4/6 (74 dB SPL) or 3/6 (88 dB SPL) of the points when measured individually (Fig. 4b).

**Fig. 5:**
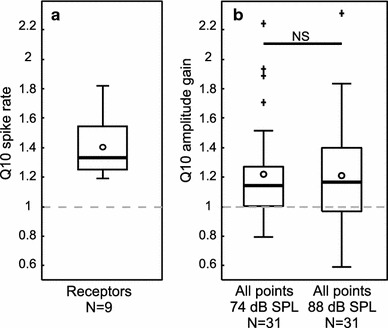
*Box plots* of calculated Q10 values for receptor neuron spike rate and tympanal membrane amplitude gain for all measured points pooled, showing median (*line*), mean (*circle*), quartiles, 1.5 IQR, and outliers (+). **a** Q10 values for spike rate of intracellularly recorded receptor neurons (*N* = 9). **b** Q10 values for amplitude gain of the tympanal membrane (*N* = 31). All six representative points located on the membrane were pooled to acquire an overall mean Q10 for each individual. There was no difference in Q10 values between the two stimulus intensities

All measured points showed mean Q10 values for amplitude gain above 1 (Fig. 4c; Table 1), indicating that tympanal membrane displacement increased in the warmer condition. There was no significant difference between Q10 values of the six measured points within one intensity (Kruskal–Wallis test −74 dB SPL: *χ* ^2^ = 4.34, NS; 88 dB SPL: *χ* ^2^ = 9.00, NS) and no significant difference between intensities (Kruskal–Wallis test: *χ* ^2^ = 13.72, NS). Amplitude gain for all points pooled together (one mean Q10 value per individual calculated over all six points) exhibited mean Q10 values of 1.22 (74 dB SPL) and 1.21 (88 dB SPL) (Fig. 5b). There was also no difference between the mean Q10 values of pooled data and intensity (Fig. 5b, Wilcoxon rank-sum test, *Z* = −0.155, NS). When taking only low (2–10 kHz) or high (12–30 kHz) sound frequencies into account, mean Q10 values did not significantly change (Table 1).

**Table 1:** Relative displacement of the tympanal membrane at 20 and 31 °C, expressed as mean Q10 values ± SD (see also Fig. 4c)

Point	Q10 (amplitude gain)	
	74 dB	88 dB
1	1.09 ± 0.43	1.06 ± 0.47
2	1.32 ± 1.04	1.22 ± 0.37
3	1.39 ± 0.69	1.28 ± 0.56
4	1.17 ± 0.34	1.33 ± 0.41
5	1.26 ± 0.49	1.08 ± 0.37
6	1.16 ± 0.35	1.34 ± 0.79
All points	1.22 ± 0.33	1.21 ± 0.35
2–10 kHz	1.21 ± 0.40	1.28 ± 0.60
12–30 kHz	1.21 ± 0.34	1.16 ± 0.28

All measured points exhibit a Q10 > 1; thus, the tympanal membrane displacement slightly increased with 10 °C increased temperature. Q10 values for all points together (one mean Q10 value calculated over all six measured points per individual) were calculated over the whole frequency range, low-frequency range (2–10 kHz) and high-frequency range (12–30 kHz)

## Discussion

Warmer temperatures lead to slightly larger tympanal membrane displacements (Figs. 4, 5) as well as to increased firing rates of auditory receptor neurons (Figs. 2, 5). In addition, the width and slope of the dynamic range and thresholds as well as the spike durations were affected (Fig. 3). We will first focus on the effects on spike rates.

The average Q10 value of 1.4 found for spike rates is remarkably low. Not only lower than the Q10 of 2–3, which is typical for various chemical, metabolic, and physiological rates across microbes, plants, and animals (Hoffmann 1995; Dell et al. 2011), but also lower than in several reports in the literature. In different insect species Q10 values for spike rates of auditory receptor neurons between 1.7 and 3.8 are reported or can be calculated from figure data (Abrams and Pearson 1982; Coro et al. 1994; Franz and Ronacher 2002; Fonseca and Correia 2007; Korsunovskaya and Zhantiev 2007). Oldfield’s (1988) data yield extreme Q10 values between 3.7 and 11.6. However, these values seem less realistic since he recorded from isolated ears (see also comments by Korsunovskaya and Zhantiev 2007). In a previous study on locusts in a similar experimental setup, a stronger temperature effect was visible, which would correspond to a Q10 of ~1.75 (Fig. 1a in Franz and Ronacher 2002). However, this value is based on the recording of a single receptor neuron and still falls in the range of Q10 values found in the present study (1.19–1.82). Differences with other authors’ reports are larger and may be attributed to differences in methods used, different species, or very large temperature ranges and a small number of receptor neurons used. The contrasting findings of our study to others might also be due to the different locust populations used; considerable variability in neurons’ physiology and temperature dependence can occur even within populations as has been shown in a study of isogenic clones of *Schistocerca americana* (Goodman and Heitler 1977).

Temperature effects on mechanosensors may also depend on body position: the sensitivity to temperature shifts of mechanosensory hairs of the locust *S. americana* changed depending on their location on the body (Miles 1985). Sensory hairs on the thorax, being subject to smaller temperature variations, reacted more sensitively to heating or cooling, while hairs on the tarsus that are normally exposed to larger temperature changes exhibited almost temperature-invariant firing rates (Miles 1985). This last observation supports our finding of low Q10 values and indicates that mechanoreceptors can evolve to respond in an almost temperature-invariant way.

Since no network effects (excitation or inhibition by other neurons) have been reported so far for insect auditory receptor neurons, their dependence on temperature must be attributed solely to the movement of the tympanal membrane, the transduction process, and intrinsic cellular processes. In particular, the activity of ion channels responsible for the generation of spikes is affected by temperature, and Q10 values for ion permeability and channel activation/inactivation for sodium, potassium, and calcium range between 1.5 and 3 (e.g. Hodgkin and Katz 1949; Montgomery and MacDonald 1990; Janssen 1992; Hille 2001). Notwithstanding the strong temperature dependence of ion channels, a recent modelling study has demonstrated that neurons can indeed achieve a low temperature dependence of firing rates with a suitable combination of ion channels (Roemschied et al. 2014).

The decrease in action potential duration with higher temperatures seen in the present study (about 0.06 ms per 1 °C) is comparable to the action potential shape changes found in other studies. In neurons of the suboesophageal ganglion of the cricket *Gryllus bimaculatus*, action potential duration decreased by approximately 0.05 ms per 1 °C in a comparable temperature range (Janiszewski and Otto 1988: see Fig. 3 therein). Two studies investigating locust auditory receptor neurons also mention a decrease in action potential duration with rising temperature, without providing quantitative data (Abrams and Pearson 1982; Franz and Ronacher 2002).

The response thresholds of auditory receptors decreased on average by only 1.6 dB and the width and slope of the dynamic range slightly increased with a temperature step of 6–8 °C. Although these trends were consistent across preparations, such small shifts in threshold or dynamic range may be of little relevance to the locust’s processing of sounds under natural and noisy conditions. In contrast, in some auditory interneurons of a cicada, *Tettigetta josei*, thresholds decreased by an average of 2.2 dB per 1 °C rise, mainly in the low-frequency range (Fonseca and Correia 2007). Wolf (1986) found a decrease in threshold with warming of 1 dB per 1 °C between 15 and 40 °C in an auditory interneuron of the grasshopper *Ch. biguttulus*. The difference to the present study may in part result from a different method used to calculate thresholds, as Wolf (1986) used a criterion: threshold = sound pressure level at which the stimulus was answered with a 50 % probability, whereas we fitted a Boltzmann function to calculate thresholds (see “Materials and methods”). Additionally, Wolf (1986) investigated an auditory interneuron, in which network effects might contribute to the neuron’s response to temperature changes.

Network effects may also reduce the impact of intrinsic temperature effects at the level of local and ascending interneurons, making intrinsic temperature compensation less important. Even if those neurons are affected by temperature, the combination of information from several neurons might result in temperature-independent codes being available to the postsynaptic network in the brain (von Helversen 1979; von Helversen and von Helversen 1994; Ronacher et al. 2004; Clemens et al. 2011, 2012; Robertson and Money 2012). In contrast, no network exists at the level of receptor neurons, whose coding abilities might thus be more affected by temperature effects. Likely, at the level of auditory interneurons other properties become more important than firing rates. Electrophysiological studies on auditory receptors and interneurons of locusts showed that the resolution of fast-amplitude modulations and the detection of minute gaps within sound stimuli were improved with rising temperature (Franz and Ronacher 2002; Prinz and Ronacher 2002; see also Ronacher and Römer 1985).

The receptors’ firing rates depend on both the intrinsic properties of the cell and the displacement of the tympanal membrane: sound waves translate to travelling waves across the tympanal membrane and physically move the receptor neurons’ attachments. Thus, enhanced tympanal displacement at higher temperatures could, at least to some extent, be responsible for the rise in spike rates observed for receptor neurons. In this study on *L. migratoria,* we found a slight increase in tympanal membrane displacement at higher temperatures (Figs. 4, 5). Since the frequency resolution of grasshoppers is rather poorly developed (Hennig et al. 2004), the present study did not focus on frequency tuning of the tympanal membrane, rather we investigated the overall movement by combining all frequencies. By using the same simple acoustic broadband stimuli as used for electrophysiology, we acquired comparable results concerning both receptor neuron physiology and tympanal membrane displacement. The mean Q10 values for displacement (amplitude gain) measured at six representative points, where four of the points were actually attachment points of Müller’s organ (Fig. 1b), were consistently above one (Fig. 4c). Still, the overall increase across all sound frequencies in movement was quite small compared to the maximal deflections of the tympanal membrane (Windmill et al. 2005, 2008).

Similar small effects of temperature changes on tympanal vibrations are reported from other insects. The tympanum and tympanal apodeme of a cicada, *T. josei*, showed no change in vibration velocity or phase angle to acoustic stimulation within a temperature range of 18–35 °C (Fonseca and Correia 2007). Also, in a tree cricket, *Oecanthus henryi*, the frequency response of the anterior tympanal membrane did not change with temperature (Mhatre et al. 2011).

Since we do not know the transfer function between tympanal membrane movement and receptor neuron spike rate in locusts, we cannot directly connect the Q10 values obtained for tympanal membrane displacement and receptor neuron spike rate. Effective stimulation of receptors results from an interaction between the resonant properties of the tympanum and the mass and elasticity of Müller’s organ (Stephen and Bennet-Clark 1982). Therefore, the effects of temperature changes on auditory mechanics alone are not clear (Mason and Faure 2004). Nevertheless, the slightly enhanced membrane displacement may at least to some extent contribute to the increased spike rate seen in auditory receptors.

For insects using acoustic signals for mate localization, species recognition, predator or prey detection, the reliable neuronal coding of such signals is crucial for an adequate behavioural reaction to sound signals. Almost temperature-independent tympanal membrane properties and a robust encoding of acoustic signals by auditory receptor neurons, whose properties change only slightly with temperature, might enable the animals to reliably identify sounds (e.g. of approaching predators) under different environmental conditions. The next steps will be to investigate second- and third-order neurons along the auditory pathway to see how these neurons are affected by changing temperatures.

## Electronic supplementary material

Below is the link to the electronic supplementary material.

Supplementary material 1a (MPG 768 kb)

Supplementary material 1b (PDF 8 kb)
