# Supplementary material for: Temperature effects on the tympanal membrane and auditory receptor neurons in the locust
Source: J Comp Physiol A Neuroethol Sens Neural Behav Physiol. 2014 Jul 22;200(9):837–47. doi: 10.1007/s00359-014-0926-y (PMC4138429; doi:10.1007/s00359-014-0926-y)
Supplement: Supplementary file 2 — Supplementary material 1b (PDF 8 kb) [file 359_2014_926_MOESM2_ESM.pdf]

Temperature effects on the tympanal membrane and auditory receptor neurons in the locust - Journal of Comparative Physiology A

Monika J.B. Eberhard*, Shira D. Gordon, James F.C. Windmill and Bernhard Ronacher

*Author for correspondence: M. J. B. Eberhard, Humboldt-Universität zu Berlin, Department of Biology, Behavioural Physiology Group, Invalidenstr. 43, 10115 Berlin, Germany
E-mail address: monika.eberhard@biologie.hu-berlin.de

Movie S1.mpg

Online Resource 1 Travelling wave across the locust, *Locusta migratoria*, tympanal membrane taken from a scanning laser vibrometer. Color indicates displacement toward the viewer (red) or away (green).
